# A Novel Ferroptosis Related Gene Signature for Prognosis Prediction in Patients With Colon Cancer

**DOI:** 10.3389/fonc.2021.654076

**Published:** 2021-05-11

**Authors:** Jianhua Nie, Dan Shan, Shun Li, Shuyuan Zhang, Xiaolin Zi, Fan Xing, Jiaqi Shi, Caiqi Liu, Tianjiao Wang, Xiaoyuan Sun, Qian Zhang, Meng Zhou, Shengnan Luo, Hongxue Meng, Yanqiao Zhang, Tongsen Zheng

**Affiliations:** ^1^ Department of Gastrointestinal Medical Oncology, Harbin Medical University Cancer Hospital, Harbin, China; ^2^ Department of Radiation Oncology, The Second Affiliated Hospital of Harbin Medical University, Harbin, China; ^3^ The Seventh Department of the Internal Medicine, Harbin Medical University Cancer Hospital, Harbin, China; ^4^ Department of Pathology, Harbin Medical University Cancer Hospital, Harbin, China; ^5^ Department of Phase 1 Trials Center, Harbin Medical University Cancer Hospital, Harbin, China; ^6^ Key Laboratory of Molecular Oncology, Heilongjiang Cancer Institute, Harbin, China

**Keywords:** ferroptosis, prognosis, colon cancer, STING, immune status

## Abstract

**Purpose:**

Colon cancer (CC) is a serious disease burden. The prognosis of patients with CC is different, so looking for effective biomarkers to predict prognosis is vitally important. Ferroptosis is a promising therapeutic and diagnosis strategy in CC. However, the role of ferroptosis in prognosis of CC has not been studied. The aim of the study is to build a prognosis model related ferroptosis, and provide clues for further therapy of CC.

**Methods:**

The RNA-seq data were from TCGA (training group) and GEO (testing group). The R language and Perl language were used to process and analyze data. LASSO regression analysis was used to build the prognosis model. ssGSEA was used to compare the immune status between two groups. Immunohistochemistry was used to detect expression of AKR1C1 and CARS1 in colon cancer tissues and adjacent tissues.

**Results:**

The prognosis model consisted of five ferroptosis related genes (AKR1C1, ALOX12, FDFT1, ATP5MC3, and CARS1). The area under curve (AUC) at 1-, 2-, and 3-year were 0.668, 0.678, and 0.686, respectively. The high- and low-risk patients had significant survival probability and could be clearly distinguished by the PCA and t-SNE analysis. The multivariate cox regression analysis also showed the riskscore is an independent prognosis factor. Importantly, we found that the immune status between high- and low-risk patients were different obviously, such as CD8^+^T cells. And STING, a new promising immune target, was also correlated to our signature genes statistically significantly.

**Conclusion:**

Our ferroptosis prognosis signature could predict survival of CC patients to a certain degree. And the crosstalk between ferroptosis and immune, especially STING need further studies.

## Introduction

According to the latest cancer epidemiology, the global incidence of colorectal cancer ranked the third (10.2%), and mortality of which ranked the second (9.2%) in both sexes combined (1, 2), leading to a huge health and economic burden. The diagnosis and treatment of colon cancer (CC) has made a progress in the advance of clinical treatments, such as immunotherapy and targeted therapy (3, 4). However, current clinical management is still far from achieving satisfying outcomes. And the prognosis of patients with CC diversifies individually. Although there are some prognostic factors, such as stage and carcinoembryonic antigen (CEA), they can’t predict patients’ prognosis accurately. We still need to search for more accurate biomarkers to predict prognosis of patients with CC, guiding clinical management as well as sparking great potential for discovering novel therapeutic targets. Therefore, there is an urgent need to find new biomarkers for CC patients.

Ferroptosis is a new recognized way of non-apoptosis regulated cell death, characterized by the iron-dependent accumulation of lipid hydroperoxides, holding great promise for fighting against cancers (5). We have already reviewed the role of ferroptosis in digestive system neoplasms in the previous paper (5), which highlighted the essential role of ferroptosis played in hepatocellular carcinoma and other digestive system neoplasms such as gastric cancer, pancreatic cancer, and CC.

A few of studies devoting to investigating the role of ferroptosis in CC proved the central role of ferroptosis in the therapy and prevention for CC. For example, triterpene saponin ardisiacrispin B and epunctanone exerted cytotoxic effects partly *via* ferroptosis in resistant HCT116 p53−/− colon adenocarcinoma cells (6, 7). By inducing ferroptosis and apoptosis, electroporation increased the sensitivity of CC cells to camptothecin analog SN38 (8). Omega-3 polyunsaturated fatty acids (n-3 PUFA) and highly fermentable fiber may induce ferroptosis to reduce the risk of colorectal cancer (9). *Betula etnensis* Raf. (Birch Etna) promoted ferroptosis mediated by heme oxygenase-1(HO-1) hyper-expression in CC (10). Bromelain exerted cytotoxic effects in Kras-mutant colorectal cancer cells *via* downregulating acyl-CoA synthetase long-chain family member 4 (ACSL4) to induce ferroptosis (11). It can be seen that induction of ferroptosis is a promising strategy in diagnosis, treatment, and prevention of CC. However, the prognosis role of ferroptosis in CC has not been assessed.

Ferroptosis is recognized as a form of immunogenic cell death (ICD), in other words, innate and adaptive immune response could be triggered by such dying cells (12). Recently, a research showed that CD8^+^ T cells regulated tumor ferroptosis during cancer immunotherapy. This is the first time that researchers have confirmed the direct crosstalk between immune system and ferroptosis (13). Immunotherapy and induction of ferroptosis are both considered to be of great significance in clinical management of colon cancer, studying the crosstalk between immunotherapy and ferroptosis thereby can be quite meaningful for developing novel treatment and overcoming resistance to immunotherapy. Stimulator of interferon genes (STING) is a new immune target in cancer therapy (14). A study showed that GPX4, a key regulatory molecule in ferroptosis, facilitated STING activation by maintaining redox homeostasis of lipids, indicating that ferroptosis was related to STING pathway. However, the direct crosstalk between STING and ferroptosis has not been studied yet.

Our study aimed to investigate the role of ferroptosis in the prognosis of CC and look for valuable targets related to ferroptosis for further experimental and clinical research, to better ameliorate clinical management of colon cancer. We also tried to provide clues for the relationship between ferroptosis and immune system, especially STING.

## Materials and Methods

### Colon Adenocarcinoma Datasets and Ferroptosis Related Genes

The RNA-seq transcriptome data and clinical data of patients with colon adenocarcinoma (COAD) were downloaded from the Cancer Genome Atlas (TCGA) database (https://portal.gdc.cancer.gov/) and Gene Expression Omnibus (GEO) database (https://www.ncbi.nlm.nih.gov/geo). We downloaded RNA-seq data of COAD cohort from TCGA as Fragments Per Kilobase of transcript per Million mapped reads (FPKM) as the training group. The RNA-seq data of GSE39582 from GEO was used as the testing group. The ferroptosis related genes were referred from previous studies (15), and the authors collected them from previous important and authoritative literatures related to ferroptosis (16–19).

### Construction of Ferroptosis Related Prognosis Model in CC

We used R language to analyze data in the present study (https://www.R-project.org/). The differentially expressed genes (DEG) related ferroptosis between normal tissues and tumor tissues were filtered by “limma” package using wilcox Test [false discovery rate (FDR) <0.05] in the TCGA cohort. Then we used the “survival” package to conduct univariate cox regression analysis (p <0.05) to filter survival-related ferroptosis genes. The intersecting genes between DEG and survival-related ferroptosis genes were used in further analysis. The Least absolute shrinkage and selection operator (LASSO) cox regression analysis (20) was used to build the ferroptosis related prognosis model in CC using “glmnet” and “survival” packages. On the basis of the results of LASSO regression analysis, riskscore of each patient was calculated by the expression levels of genes and the corresponding coefficient in prognosis model using the following formula: Riskscore = Exp_gene 1*_ Coef_gene 1 +……_Exp_gene n*_ Coef_gene n_, with Coef indicating the coefficient and Exp indicating the expression level of genes. Then all patients were divided into high-risk groups (whose riskscores are above median values) and low-risk groups (whose riskscores were below median values) based on the riskscore.

### The Verification of Ferroptosis Related Prognosis Model in CC

Receiver operating characteristic (ROC) was used to evaluate the accuracy of our model using the “survival,” “survminer,” and “timeROC” packages. The Kaplan-Meier curve was used to compare the survival probability difference between high-risk groups and low-risk groups using the log-rank test with “survival” and “survminer” packages. Principal component analysis (PCA) and t-distributed stochastic neighbor embedding (t-SNE) analysis were used to visualize the data in two dimensions with the “Rtsne” and “ggplot2” packages. The univariate and multivariate Cox regression analysis were used to determine the independent prognosis factors using the “survival” packages. A nomogram was used to predict survival probability of CC patients with the R package “rms.”

### The Function Analysis of Ferroptosis Related Prognosis Model in CC

Gene ontology (GO) and Kyoto Encyclopedia of Genes and Genomes (KEGG) were conducted to discover the potential function of genes between high- and low-risk groups. Single-sample gene set enrichment analysis (ssGSEA) was used to evaluate the infiltration levels of immune cell types and immune function between the high- and low-risk groups in the R packages of “gsva” (21). The correlation between ferroptosis genes and STING related genes in CC was evaluated using Pearson’s correlation coefficient.

### Protein-Protein Interactions (PPI) Network and Correlation Plot

The possible predicted pathways between ferroptosis related molecules and STING related molecules were indicated by the correlation plot and PPI network from the perspective of RNA and protein levels, respectively. We built the PPI network by online STRING database (https://string-db.org/). When inputing multiple proteins by names in the search bar, then PPI network was built automatically. We built the correlation plot through “igraph” and “reshape2” packages.

### The Human Protein Atlas (HPA)

The human protein atlas (HPA) (22, 23) is a free database which consists of multiple protein expression images in normal tissues and cancer tissues. The immunohistochemistry images of the corresponding genes in the prognosis model were searched in the HPA database to verified the bioinformatics analysis results in our study.

### Immunohistochemistry

To verify the results of this study, we collected 54 paired tumor tissues and adjacent tissues from our hospital to carry out immunohistochemistry. Immunohistochemistry was performed as described previously (24, 25) using anti-AKR1C1(ab192785, Abcam) and anti-CARS1(ab126714, Abcam) antibodies. In brief, colon cancer tissues and adjacent tissues sections were deparaffinized in xylene and rehydrated with ethanol. Heat mediated antigen retrieval was performed in 10 mM citrate buffer at pH 6.0. After blocking with normal goat serum, slides were incubated with primary antibodies against AKR1C1 and CARS1 overnight (4°C). Tissue sections were then stained with biotinylated secondary antibody (Vector lab) for 1 h at room temperature, followed by the Vectastain Elite ABC reagent (Vector lab) for 30 min. The peroxidase reaction was developed with diaminobenzidine (DAB kit; Vector lab) and the slides were counterstained with hematoxylin (Sigma).

The positive expression of AKR1C1 and CARS1 were mainly located in the cytoplasm. Positive cells accounted for a percentage score standard: 0 = no positive cells; 1 = 1–25% positive cells; 2 = 26–50% positive cells; 3 = 51–75% positive cells; 4 = more than 75% positive cells.

## Results

### Establishment of Ferroptosis Related Prognosis Signature in Colon Cancer

**Figure 1:**
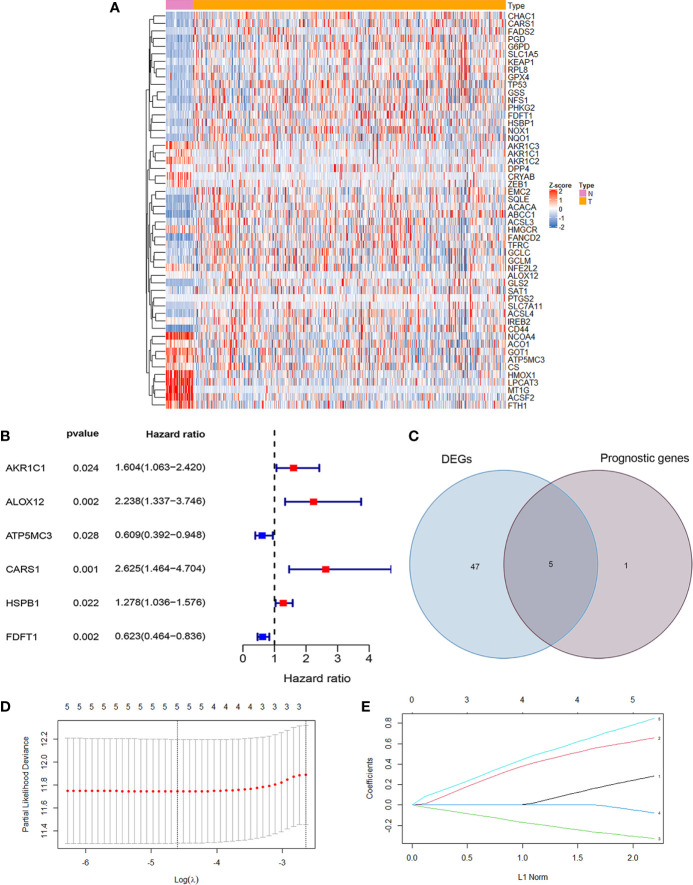
Development of ferroptosis related gene signature in colon cancer. **(A)** The heatmap showed the differentially expressed genes (DEG) related ferroptosis between normal population and colon cancer population from TCGA (P <0.05). **(B)** The forest map showed six prognosis related genes in colon cancer by univariate cox regression analysis (P <0.05). **(C)** The Venn diagram showed the intersecting genes between DEG and survival related genes. **(D)** Partial likelihood deviance against log (λ) is plotted. The first vertical dashed line representatives the λ value with minimum error. **(E)** The LASSO coefficient profiles of ferroptosis-related gene in colon cancer.

The RNA-seq data and clinical data in patients with CC were from two public databases, TCGA and GEO. The ferroptosis related differentially expressed genes between normal population (n = 41) and colon cancer population (n = 473) from TCGA were showed in **Figure 1A**. The prognosis related genes *via* univariate Cox analysis were presented in **Figure 1B**, which implicated AKR1C1, ALOX12, CARS1, and HSPB1 were risk genes for the prognosis of colon cancer patients. However, ATP5MC3 and FDFT1 were protective genes for colon cancer patients. The intersecting genes between differentially expressed ferroptosis related genes and survival related genes were AKR1C1, ALOX12, CARS1, FDFT1, and ATP5MC3 (**Figure 1C**). Next, we built a ferroptosis related gene signature using the above five genes with the method of LASSO regression analysis (**Figures 1D, E**). The riskscore was calculated for each CC patients as the following formula:

0.281948648331575 *the expression levels of AKR1C1 + 0.655775537737114 *the expression levels of ALOX12 + 0.845349664371856 *the expression levels of CARS1 + (−0.327305379054264) *the expression level of FDFT1 +(−0.0769643246539205)*the expression levels of ATP5MC3. To sum up, we have built a ferroptosis related prognosis model to predict prognosis of colon cancer patients, which needed further verification.

### Verification of Accuracy of Five Ferroptosis Related Gene Signature in Colon Cancer

**Figure 2:**
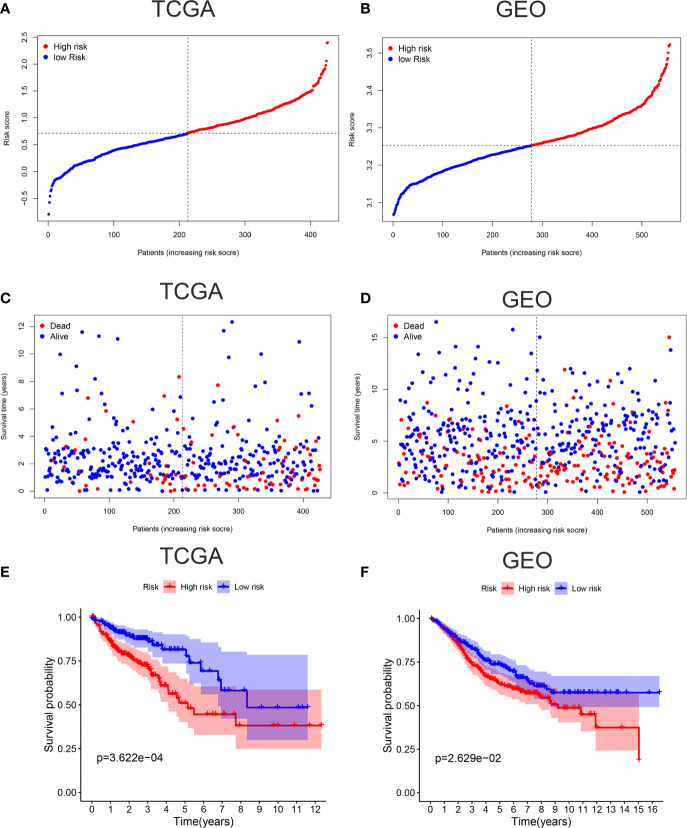
Verification of accuracy of ferroptosis related gene signature in colon cancer. **(A)** The patients from TCGA cohort are divided into high-risk group and low-risk group based on the riskscore median values. **(B)** The patients from GEO cohort are divided into high-risk group and low-risk group based on its own riskscore median values. **(C)** The distribution of survival time in the high-risk group and low-risk group in the TCGA cohort. **(D)** The distribution of survival time in the high-risk group and low-risk group in the GEO cohort. **(E)** Kaplan-Meier curves showed the survival differences between high-risk group and low-risk group using the log-rank test in the TCGA cohort. **(F)** Kaplan-Meier curves showed the survival differences between high-risk group and low-risk group using the log-rank test in the GEO cohort.

To verify the accuracy of our model, Kaplan-Meier survival curves, ROC curves, PCA, and t-SNE were plotted in TCGA cohort and GEO cohort respectively. All patients were divided into high-risk groups and low-risk groups based on the respective median values from TCGA (**Figure 2A**) and GEO database (**Figure 2B**). **Figures 2C, D** showed the survival time trends of all the patients with the increasing of riskscore. The red dots represent the dead patients, and the blue dots represent the patients still be alive. We found that the higher the riskscore was, the more dead people were, proving that our model could predict prognosis of CC patients to a certain degree. In addition, we used a chi-square test to compare the number of dead and surviving patients between the high- and low-risk groups in two cohorts. The results indicated that there were more dead patients in the high-risk groups (P <0.01) (**Supplementary Figure 1**). The Kaplan-Meier survival curves showed that the high-risk groups were less likely to survive than the low-risk group in TCGA cohort (p <0.001) and GEO cohort (P <0.05) (**Figures 2E, F**).

**Figure 3:**
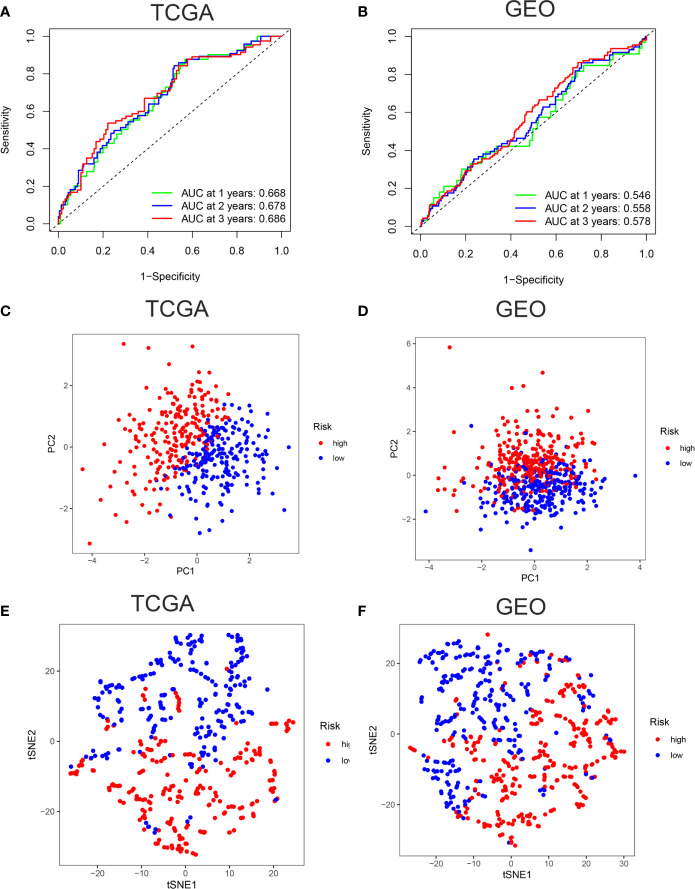
Verification of accuracy of ferroptosis related gene signature in colon cancer. **(A)** The AUC score at 1, 2, 3 years in the TCGA cohort. **(B)** The AUC score at 1, 2, 3 years in the GEO cohort. **(C)** The PCA plot in the TCGA cohort. **(D)** The PCA plot in the GEO cohort. **(E)** The t-SNE plot in the TCGA cohort. **(F)** The t-SNE plot in the GEO cohort.

ROC curves showed the AUC scores in the 1, 2, and 3 years were 0.668, 0.678, and 0.686, respectively in TCGA cohort (**Figure 3A**). In the GEO cohort, AUC scores in the 1, 2, and 3 years were 0.546, 0.558, and 0.578, respectively (**Figure 3B**). PCA and t-SNE analysis were also applied to test the accuracy of our prognosis model. The PCA plot and t-SNE plot indicated that patients in the high-risk and low-risk group were in two directions in the TCGA and GEO cohort (**Figures 3C–F**). Overall, our five ferroptosis related genes signature can distinguish the prognosis of colon cancer patients to some extent.

What’s more, to further confirm the accuracy of the model, we did Kaplan-Meier survival analysis, univariate Cox regression analysis, and LASSO regression analysis towards three typical DEGs (CD44, GCLC, and MT1G). The results implicated that CD44, GCLC, and MT1G were not correlated to prognosis of patients (P>0.05). The Partial likelihood deviance and LASSO coefficient profiles showed CD44, GCLC, and MT1G were not suitable as the model genes (**Supplementary Figure 2**).

### Riskscore and Other Clinical Pathology Characters Synergistically Predicted the Survival Probability of Colon Cancer Patients

**Figure 4:**
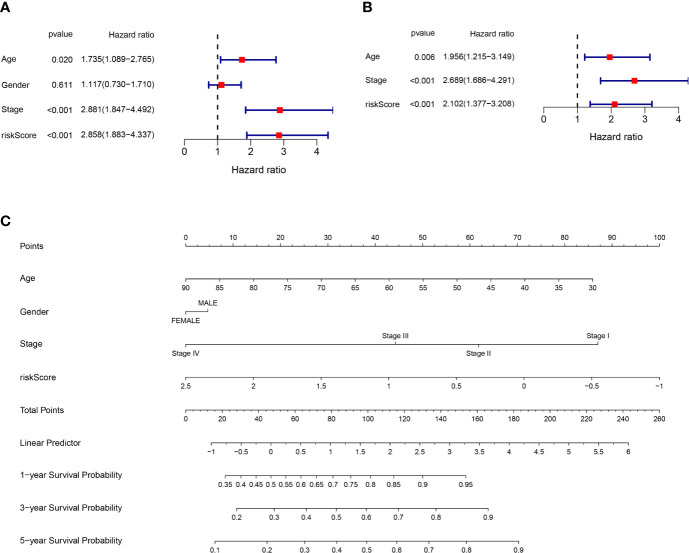
Riskscore and other clinical pathology characters synergistically predicted the survival probability of colon cancer patients. **(A)** Univariate cox regression analysis of age, gender, stage, and riskscore in the TCGA cohort. Riskscore is significantly associated with the survival of colon cancer patients. **(B)** Multivariate cox regression analysis of age, stage, and riskscore in the TCGA cohort. Riskscore is an independent prognostic factor for the survival of colon cancer patients. **(C)** Nomogram for the prediction of 1-, 3-, and 5-year survival probability in patients with colon cancer.

Univariate and multivariate Cox regression analysis were applied to test whether riskscore is an independent prognostic factor for overall survival (OS). Age, stage, and riskscore were statistically significant associated with the prognosis of colon cancer in TCGA cohort through univariate cox regression analyses, especially riskscore (HR = 2.858, 95% CI = 1.883–4.337, P<0.001) (**Figure 4A**). **Figure 4B** showed that age, stage, and riskscore were independent prognostic factor using multivariate cox regression analyses for OS in the TCGA cohort (HR = 2.102, 95% CI = 1.377–3.208, P<0.001). The univariate and multivariate cox regression analyses of GEO cohort were presented in **Supplementary Figure 3** (P<0.05). We know that single character is not good enough to predict the prognosis of CC patients, so we try to use the multi-characters to better predict their prognosis. Nomogram is a powerful tool to predict prognosis, which may be helpful for the doctors and patients. The system includes four indexes: age, gender, stage, and riskscore. For a specific colon cancer patient, his/her age, gender, stage, and riskscore have different scores, then the sum of four points is used to predict the 1-, 3-, and 5-year survival probability (**Figure 4C**). In short, riskscore was an independent prognostic factor and synergistically predicted the survival probability of CC patients.

### The Immune Status Difference Between High-Risk and Low-Risk Colon Cancer Patients

**Figure 5:**
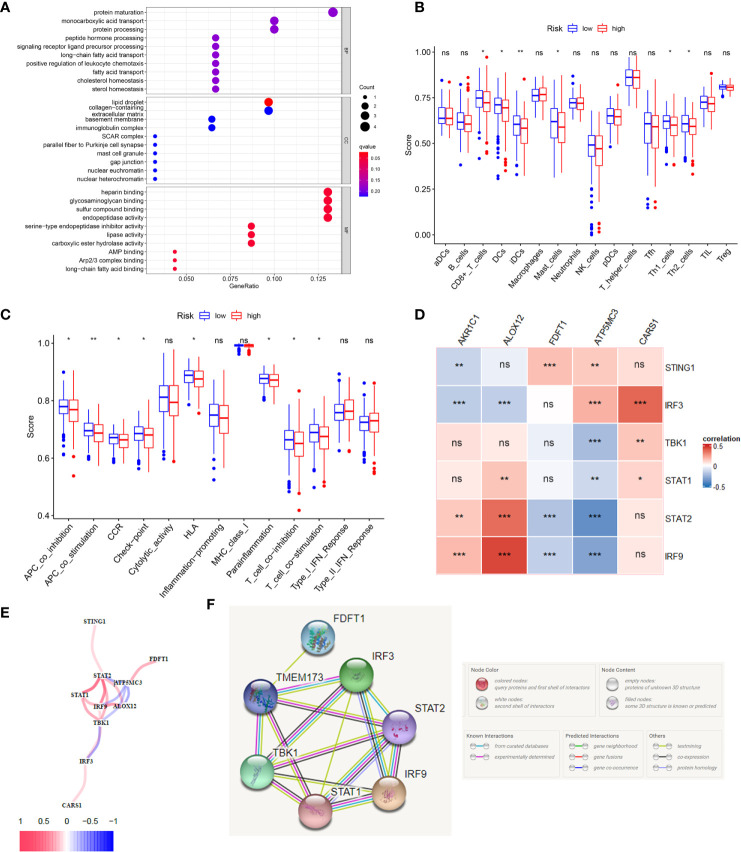
The immune status difference between high risk and low risk in colon cancer patients. **(A)** The GO enrichment analysis between high-risk and low-risk groups in colon cancer. **(B)** The immune cell between high-risk and low-risk groups in colon cancer; *P <0.05; **P <0.01; ***P <0.001. **(C)** The immune related function between high-risk and low-risk groups in colon cancer; *P <0.05; **P <0.01; ***P <0.001. **(D)** The correlation between STING related genes and ferroptosis related genes in CC using the Pearson coefficient; *P <0.05; **P <0.01; ***P <0.001; ns, no significance. **(E)** The predicted gene interactions between STING related genes and ferroptosis related genes based on the RNA-seq data of TCGA cohort in CC. **(F)** The protein-protein interactions (PPI) network of above genes.

According to the riskscore for each patient, we preformed GO and KEGG enrichment analysis between high-risk and low-risk groups. The GO result showed the differences between two groups mainly focus on fatty acid transport, positive regulation of leukocyte chemotaxis, protein maturation, mast cell granule and immunoglobulin complex (**Figure 5A**), which gave us a hint that the differences between two groups were related to immune response, so we further carried out the ssGSEA to compare immune cells and immune function differences between two groups. KEGG results were showed in **Supplementary Figure 4**. **Figure 5B** showed immature dendritic cells (iDCs) were distinct between high- and low-risk groups (P <0.01). In addition, CD8^+^T cells, DCs, mast cells, Th1 cells, and Th2 cells were different in two groups (P <0.05). Immune related functions such as APC co stimulation, APC co inhibition, CCR, checkpoint, T cell co stimulation, T cell co inhibition, and so on, were also different in two groups (**Figure 5C**). In summary, the immune status in high-risk and low-risk groups were totally different, which can be further elucidated to boost tumor immunotherapy in colon cancer. We used the Pearson’s correlation coefficient to grasp the relationship between STING and ferroptosis as showed in **Figure 5D**. The predicted pathways between STING related genes and our model genes based on the TCGA data were showed in **Figure 5E**. **Figure 5F** presented the possible protein-protein interactions between STING and ferroptosis at the protein level.

### Experimental Support for the Five Gene Prognostic Signature in Colon Cancer

**Figure 6:**
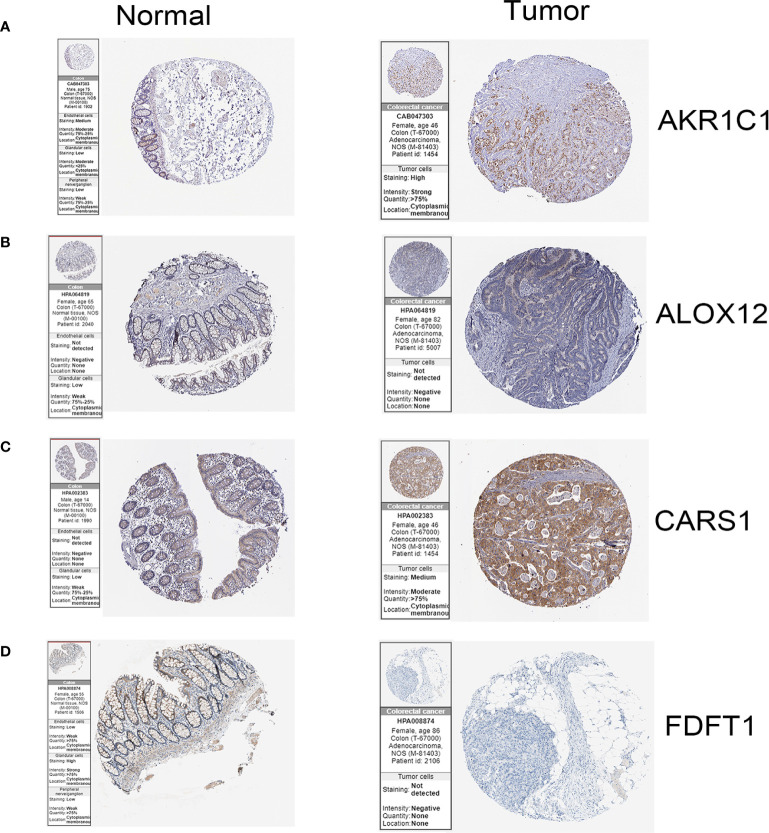
The immunohistochemistry images of related genes from HPA database in normal and cancer tissues.**(A)** The expression levels of AKR1C1 in normal tissues and colon cancer tissue. **(B)** The expression levels of ALOX12 in normal tissues and colon cancer tissue. **(C)** The expression levels of CARS1 in normal tissues and colon cancer tissue. **(D)** The expression levels of FDFT1 in normal tissues and colon cancer tissue.

**Figure 7:**
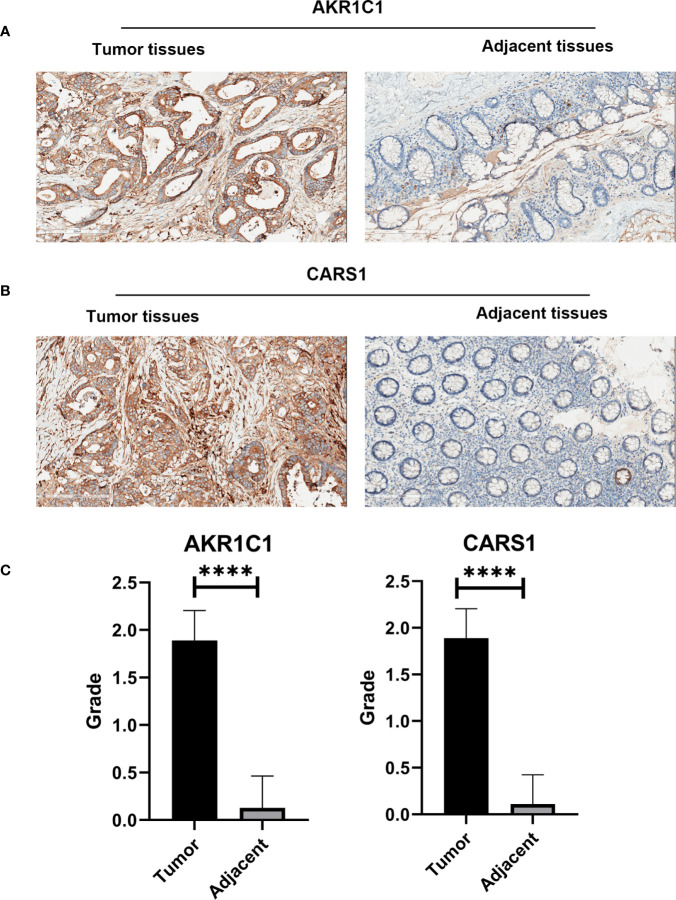
The immunohistochemistry images from wet lab. **(A)** The representative images of AKR1C1 in the tumor tissues and adjacent tissues. **(B)** The representative images of CARS1 in the tumor tissues and adjacent tissues. **(C)** The statistical results of expression of AKR1C1 and CARS1 in the tumor tissues and adjacent tissues; ****P <0.0001.

In the HPA database, we found that the expression of AKR1C1, ALOX12, and CARS1 in colon cancer tissue is higher than normal tissue (**Figures 6A–C**) and the expression of FDFT1 in colon cancer tissue is lower than normal tissue by immunohistochemistry (**Figure 6D**), which is consistent with our results. We also performed immunohistochemistry against AKR1C1 and CARS1 in the colon cancer tissues and adjacent tissues. The representative images of immunohistochemistry were indicated in **Figures 7A**, **B**. **Figure 7C** showed the expression of AKR1C1 and CARS1 were higher in tumor tissues (****, P<0.0001).

## Discussion

Our study built a ferroptosis related genes prognosis model in CC, which aimed to predict survival probability of patients with CC and provide clues for further studying the role of ferroptosis in colon cancer. The model consisted of five genes, AKR1C1, ALOX12, CARS1, FDFT1, and ATP5MC3.

Based on the DEG and prognosis related genes in CC, we selected five genes to conduct LASSO regression analysis. And all the five genes were included in the terminal model with corresponding coefficients, then we calculated the riskscore of each CC patients according to the previously mentioned formula. The Kaplan-Meier curves indicated the statistically significant survival probability between the high-risk and low-risk groups in TCGA and GEO cohort, which preliminary proofed the validity of the model to predict the risk in CC. What’s more, the AUC at 1, 2 and 3 years were 0.668, 0.678, and 0.686 respectively in TCGA cohort, which further illustrated the accuracy of the model. However, the AUC in GEO cohort was not optimal, which might attribute to the heterogeneity of patients and needs other larger population to test the model. PCA and t-SNE analysis demonstrated that the patients in high- and low-risk groups were in two dimensions respectively, indicating that the model could accurately distinguish high- and low-risk patients. Furthermore, the riskscore was an independent prognostic factor in CC. The nomogram combined the known risk factors and riskscore to predict the survival probability at 1, 3, and 5 years of CC, whose prognosis efficacy was prior to the single factor. Immunohistochemistry images of the four genes from HPA database were consistent with the bioinformatics results. We also collected 54 pairs of the tumor issues and adjacent tissues to perform IHC. Results showed the expression of AKR1C1 and CARS1 were different in tumor tissues and adjacent tissues. In summary, our model is a good signature to help to predict the prognosis of CC patients.

Aldo-keto reductases (AKRs) play vital roles in the reductive metabolism (26). The overexpression of AKR1C1 involved in the resistance of cis-diamminedichloroplatinum (CDDP) in colon cancers (27). In melanoma, upregulated AKR1C1 resulted in ferroptotic cell death resistance (28), which perhaps could explain the CDDP resistance in colon cancer and pointed out a possible strategy to overcome CDDP resistance in CC. Besides, AKR1C1 inhibitors could be a therapy medicine to sensitive ferroptosis in colon cancer. Genetic variability in arachidonate lipoxygenase (ALOXs), such as the polymorphisms of ALOX12, may influence risk of colorectal cancer (29). ALOX12 inactivation eliminated p53-mediated ferroptosis in Eμ-Myc lymphoma models (30). Thus, ALOX12 can be a possible target to regulate risk of colon cancer. A study showed that downregulation of FDFT1 was correlated with malignant progression and poor prognosis in colorectal cancer. Somatic variants revealed FDFT1 that frequently mutated only in the liver metastatic patients and targeting FDFT1 could be a feasible strategy in colon cancer, especially in the colorectal liver metastatic patients (31). To sum up, the genes in our model played important roles in colon cancer and were worthy for further studies.

To clarify the function of our model deeply, we conducted GO and KEGG enrichment analysis. It was worth noting that differential genes between high- and low-risk groups were related to immune response, which was consistent with the above phenomenon regarding to the crosstalk between immune and ferroptosis. Then we conducted the ssGSEA to compare the immune cells and immune related function in two groups. According to the results, the innate and adaptive related cells and process were statistically significant in two groups, such as CD8^+^T cells, iDC, and APC co stimulation, which perhaps presented that targeting ferroptosis could change the immune status in colon cancer or boost the immunotherapy in colon cancer. Our previous study showed that STING is a master regulator in the cancer-immunity cycle (14). In this study, we studied the relationship between STING and ferroptosis in CC. We found that the STING related genes were interrelated with ferroptosis in the levels of genes and proteins. However, the exact crosstalk and mechanism of above bioinformatics prediction need verification with well-designed experiments.

In conclusion, we built a prognosis model based on ferroptosis related genes in colon cancer with good prognosis efficacy. We also provide clues to further explore the function of the model genes and crosstalk between immune and ferroptosis to improve immunotherapy for colon cancer.

## Data Availability Statement

The original contributions presented in the study are included in the article/**Supplementary Material**. Further inquiries can be directed to the corresponding authors.

## Ethics Statement

All patients in this study have provided written informed consent for the use of their specimens and information for future investigations according to guidelines of the ethics committee.

## Author Contributions

TZ, YZ, and JN offered main direction and significant guidance of this manuscript. JN, DS, SL, SZ, XZ, FX, JS, CL, TW, XS, MZ, QZ, and SNL analyzed data and illustrated the figures for the manuscript. HM performed the immunohistochemistry. JN, SL, and SZ wrote the paper with input from all authors. All authors contributed to the article and approved the submitted version.

## Funding

This work was supported by ‘Tou Yan’ Action of Heilongjiang province, the National Youth Talent Support Program of China (W03070060), the National Natural Science Foundation of China (No. 81872435, No. 81672930 and No. U20A20377 to TZ), and the Excellent Youth Foundation of Heilongjiang Province (No. JQ2019H003). The funders had no role in study design, data collection and analysis, decision to publish, or preparation of the manuscript.

## Conflict of Interest

The authors declare that the research was conducted in the absence of any commercial or financial relationships that could be construed as a potential conflict of interest.

## Supplementary Material

Supplementary Figure 1 The comparison of the number of dead and alive patients between the high- and low-risk groups in two cohorts. Two bar charts indicated that the numbers of dead and alive patients were statistical different in the high and low risk groups in the TCGA (P<0.001) and GEO cohorts (P<0.01). This figure is related to **Figures 2C, D**.

Supplementary Figure 2 The survival plot, forest map and Lasso results of CD44, GCLC and MT1G in colon cancer. **(A–C)** The expression of CD44, GCLC and MT1G were not related to survival in colon cancer. **(D)** The forest map showed CD44, GCLC and MT1G were not prognostic factors in colon cancer. **(E, F)** The partial likelihood deviance and LASSO coefficient profiles of CD44, GCLC and MT1G were showed. Results showed CD44, GCLC and MT1G should not be included in the model, although they were significant DEGs.

Supplementary Figure 3 The riskscore was an independent prognostic factor for colon cancer patients in the GEO cohort. **(A)** Univariate cox regression analysis of age, gender, stage and riskscore in the GEO cohort. Riskscore is significantly associated with the survival of colon cancer patients (P<0.01). **(B)** Multivariate cox regression analysis of age, stage and riskscore in the GEO cohort. Riskscore is an independent prognostic factor for the survival of colon cancer patients (P<0.01).

Supplementary Figure 4 The KEGG enrichment analysis in colon cancer. The KEGG enrichment analysis between high-risk and low-risk groups in colon cancer.
