# Supplementary figures and images for: A Novel Ferroptosis Related Gene Signature for Prognosis Prediction in Patients With Colon Cancer
Source: Front Oncol. 2021 May 11;11:654076. doi: 10.3389/fonc.2021.654076 (PMC8144717; doi:10.3389/fonc.2021.654076)

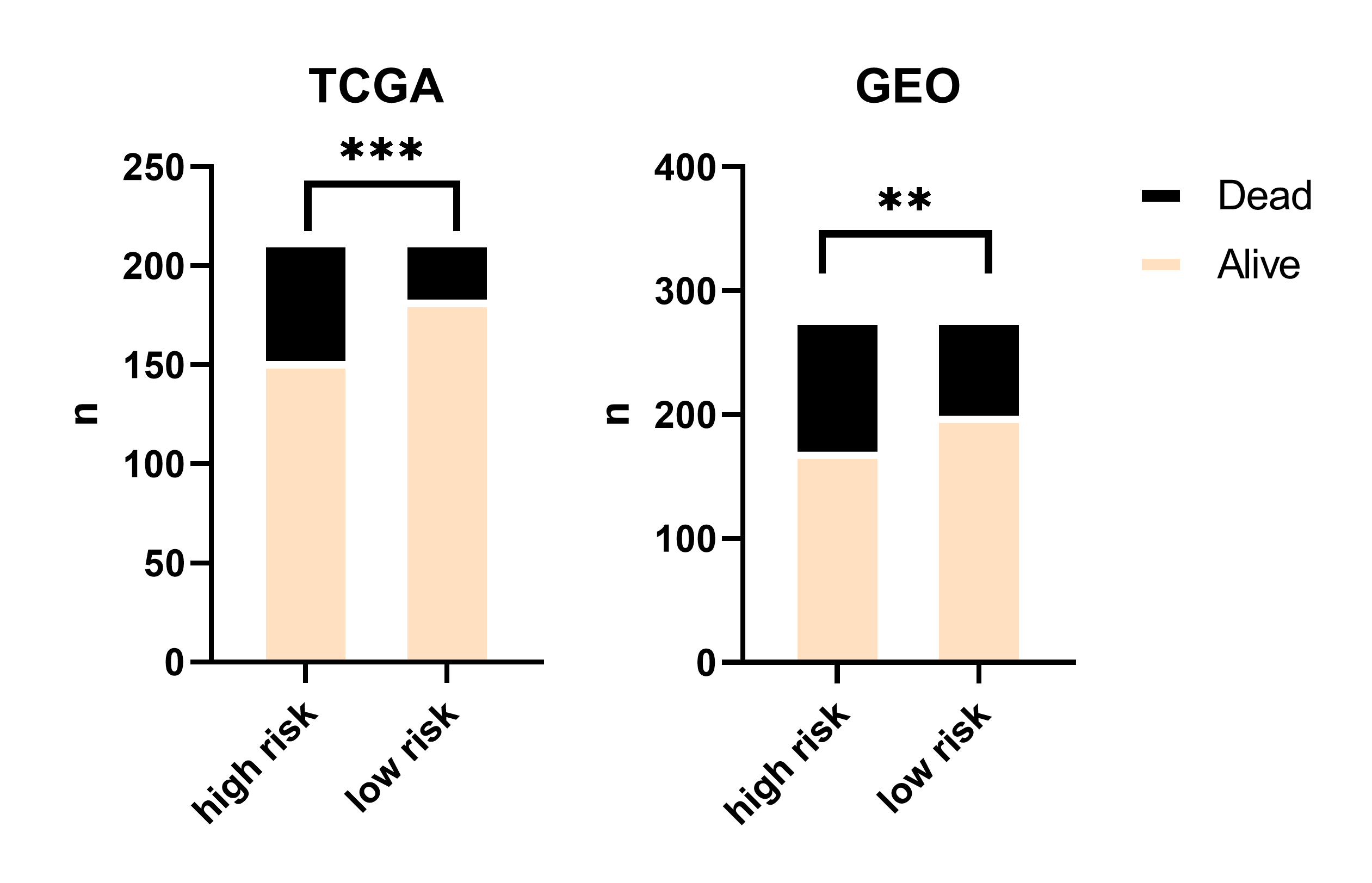

Supplement: Supplementary Figure 1 — The comparison of the number of dead and alive patients between the high- and low-risk groups in two cohorts. Two bar charts indicated that the numbers of dead and alive patients were statistical different in the high and low risk groups in the TCGA (P<0.001) and GEO cohorts (P<0.01). This figure is related to Figures 2C, D . [file Image_1.tif]

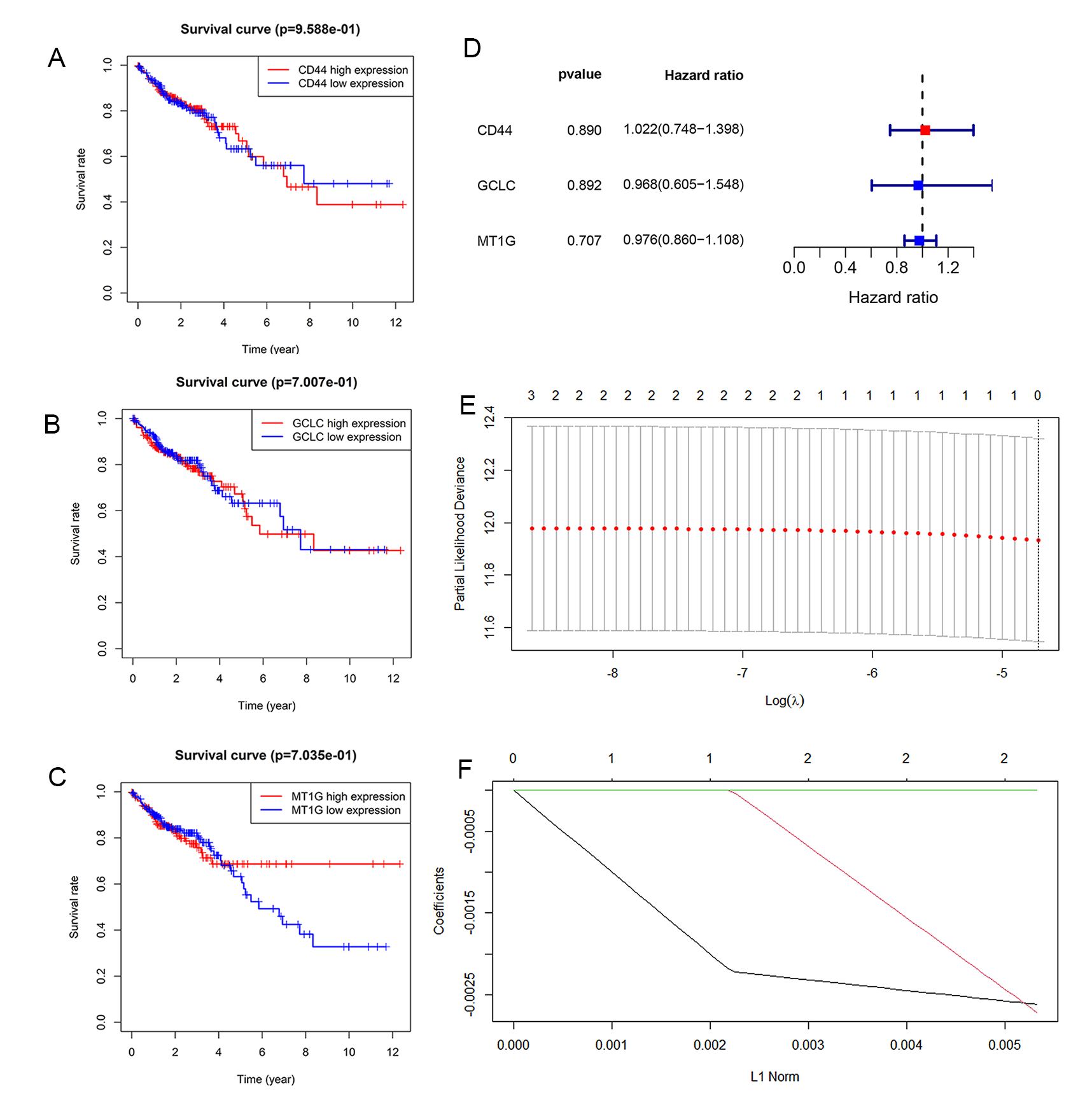

Supplement: Supplementary Figure 2 — The survival plot, forest map and Lasso results of CD44, GCLC and MT1G in colon cancer. (A–C) The expression of CD44, GCLC and MT1G were not related to survival in colon cancer. (D) The forest map showed CD44, GCLC and MT1G were not prognostic factors in colon cancer. (E, F) The partial likelihood deviance and LASSO coefficient profiles of CD44, GCLC and MT1G were showed. Results showed CD44, GCLC and MT1G should not be included in the model, although they were significant DEGs. [file Image_2.tif]

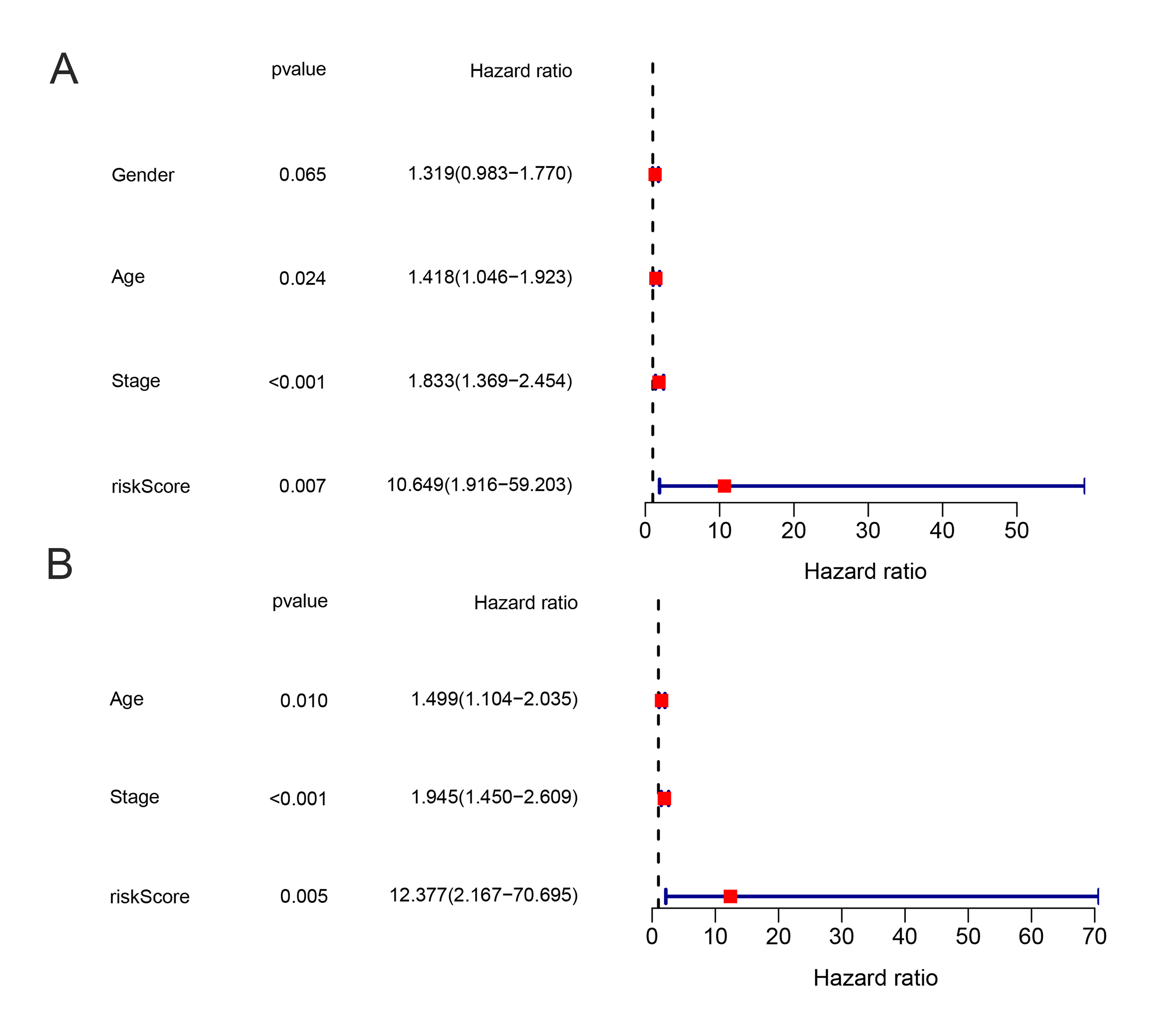

Supplement: Supplementary Figure 3 — The riskscore was an independent prognostic factor for colon cancer patients in the GEO cohort. (A) Univariate cox regression analysis of age, gender, stage and riskscore in the GEO cohort. Riskscore is significantly associated with the survival of colon cancer patients (P<0.01). (B) Multivariate cox regression analysis of age, stage and riskscore in the GEO cohort. Riskscore is an independent prognostic factor for the survival of colon cancer patients (P<0.01). [file Image_3.tif]

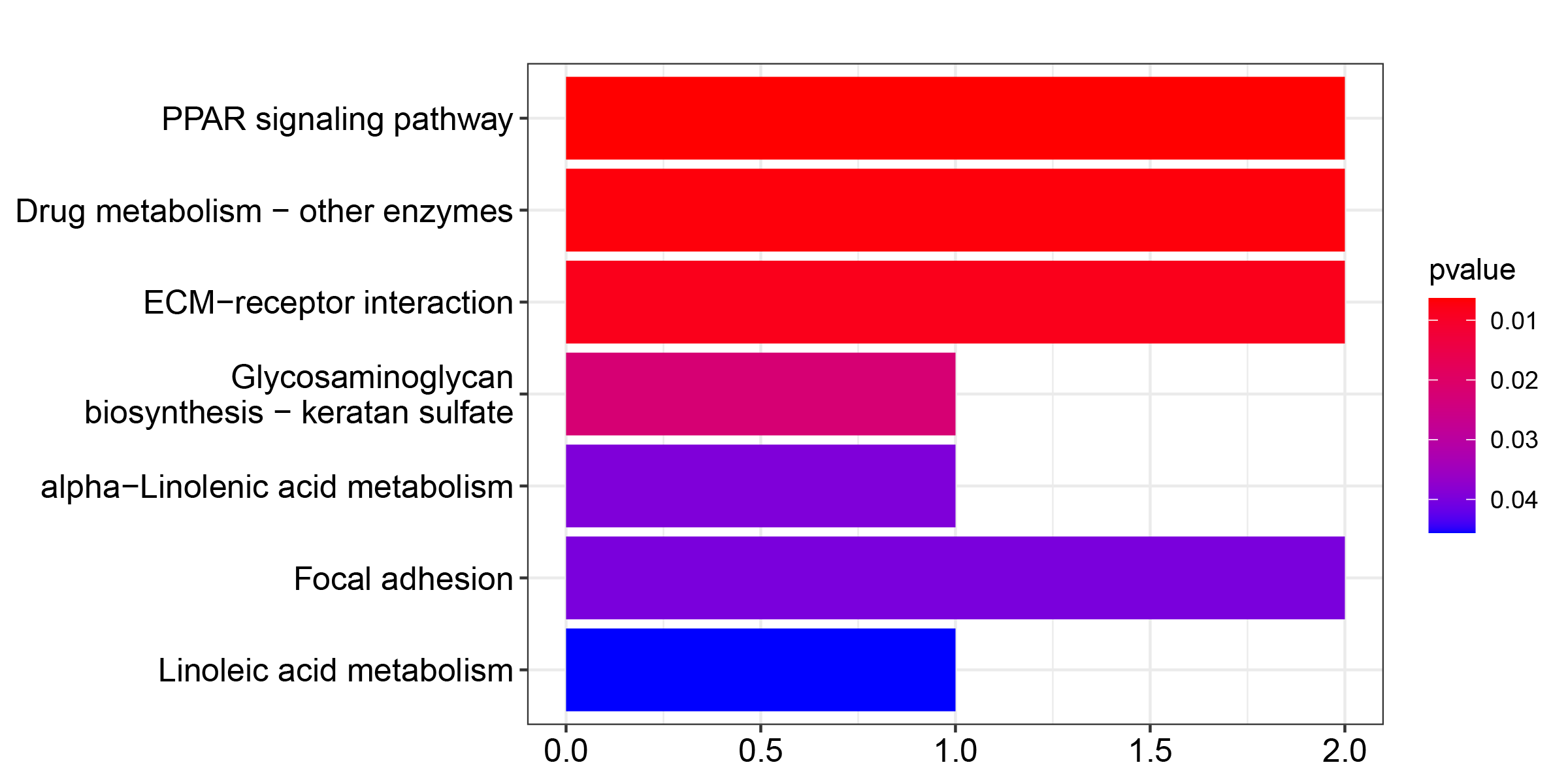

Supplement: Supplementary Figure 4 — The KEGG enrichment analysis in colon cancer. The KEGG enrichment analysis between high-risk and low-risk groups in colon cancer. [file Image_4.tif]
